# Diminished Condensin Gene Expression Drives Chromosome Instability That May Contribute to Colorectal Cancer Pathogenesis

**DOI:** 10.3390/cancers11081066

**Published:** 2019-07-28

**Authors:** Allison K. Baergen, Lucile M. Jeusset, Zelda Lichtensztejn, Kirk J. McManus

**Affiliations:** 1 Department of Biochemistry & Medical Genetics, University of Manitoba, Winnipeg, MB R3E 0J9, Canada; 2 Research Institute in Oncology & Hematology, CancerCare Manitoba, Winnipeg, MB R3E 0V9, Canada

**Keywords:** condensin, chromosome instability, colorectal cancer, single cell quantitative imaging microscopy, micronucleus

## Abstract

Chromosome instability (CIN), or constantly evolving chromosome complements, is a form of genome instability implicated in the development and progression of many cancer types, however, the molecular determinants of CIN remain poorly understood. Condensin is a protein complex involved in chromosome compaction, and recent studies in model organisms show that aberrant compaction adversely impacts mitotic fidelity. To systematically assess the clinical and fundamental impacts that reduced condensin gene expression have in cancer, we first assessed gene copy number alterations of all eight condensin genes. Using patient derived datasets, we show that shallow/deep deletions occur frequently in 12 common cancer types. Furthermore, we show that reduced expression of each gene is associated with worse overall survival in colorectal cancer patients. To determine the overall impact that reduced condensin gene expression has on CIN, a comprehensive siRNA-based screen was performed in two karyotypically stable cell lines. Following gene silencing, quantitative imaging microscopy identified increases in CIN-associated phenotypes, including changes in nuclear areas, micronucleus formation, and chromosome numbers. Although silencing corresponded with increases in CIN phenotypes, the most pronounced phenotypes were observed following *SMC2* and *SMC4* silencing. Collectively, our clinical and fundamental findings suggest reduced condensin expression and function may be a significant, yet, underappreciated driver of colorectal cancer.

## 1. Introduction

Colorectal cancer (CRC) is the second leading cause of cancer-related deaths in North America with approximately 170,000 new diagnoses and approximately 60,000 individuals succumbing to the disease each year [1,2]. The development of novel therapeutic strategies aimed at minimizing CRC morbidity and mortality rates requires novel insight into the aberrant genes and pathways driving disease pathogenesis. In this regard, chromosome instability (CIN) is a predominant form of genome instability found in many cancer types, but it is arguably best understood in CRC contexts. CIN is defined as an increase in the rate at which whole chromosomes, or large parts thereof, are gained or lost, and is found in up to 85% of all CRCs [3]. Two non-mutually exclusive forms of CIN exist: (1) Numerical CIN (N-CIN), which is associated with changes in chromosome numbers, and (2) Structural CIN (S-CIN), which is associated with structural changes (e.g., large deletions, insertions, and translocations) in chromosomes. Biochemical and genetics studies have revealed that CIN often arises due to defects in DNA replication, DNA repair, centrosome duplication, sister chromatid cohesion, kinetochore-microtubule attachments, mitotic spindle dynamics, and chromosome segregation, which are all drivers of genetic, cellular, and intratumoral heterogeneity (reviewed in [4,5,6]). As a result, CIN is synonymous with cell-to-cell heterogeneity, which manifests as diverse chromosome (i.e., genetic) complements that presumably confer distinct growth/selective advantages. As a result, CIN is implicated in cancer development and progression, and is associated with cellular transformation [7], intratumoral heterogeneity [8], metastatic progression [9], the acquisition of drug resistance [10], and poor patient prognosis [9,11]. Despite these associations, our fundamental understanding of the aberrant genes and pathways giving rise to CIN, remains poor. Of the approximately 2300 CIN genes (i.e., those whose diminished expression/function drives CIN) predicted to exist within the human genome through cross-species approaches [12], fewer than 150 have been identified and validated to date. Thus, studies aimed at identifying and characterizing novel CIN genes and their associated pathways will greatly expand our limited understanding of the aberrant molecular events underlying CIN and cancer, and may also identify candidate genes with potential diagnostic, prognostic or therapeutic value.

Over the past several decades, studies performed in a diverse array of model organisms have shown that aberrant mitotic chromosome compaction is associated with chromosome missegregation and reduced mitotic fidelity [13,14,15]. Despite these seminal observations, the aberrant genes and pathways giving rise to these abnormal phenotypes remain largely unknown. Condensin is an evolutionarily conserved protein complex that regulates chromosome compaction (reviewed in [16,17]), but its impact on CIN and cancer has never been systematically evaluated in a cancer context. In humans, two pentameric condensin complexes exist, condensin I and condensin II, which are both comprised of three domains: (1) the structural maintenance of chromosome (SMC) domain comprised of SMC2 and SMC4 found in both condensin I and II; (2) the kleisin domain containing either NCAPH (condensin I) or NCAPH2 (condensin II); and (3) the HEAT (Huntingtin, elongation factor 3, protein phosphatase, and yeast kinase TOR1 domains) domain containing NCAPD2/NCAPG (condensin I) or NCAPD3/NCAPG2 (condensin II) [18]. Both complexes are key structural components of chromosomes that function to condense/compact chromosomes during mitosis and facilitate sister chromatid disentanglement [18,19]. Interestingly, reduced expression of several condensin complex members has been investigated in a variety of model systems and is associated with aberrant chromosome compaction and chromosome missegregation [20,21,22], suggesting reduced expression of all condensin genes may drive CIN and contribute to cancer pathogenesis.

In this study, we explored the relationship between hypomorphic expression of all eight condensin genes, CIN and its implications in cancer pathogenesis. Using publicly available patient-derived datasets from The Cancer Genome Atlas (TCGA) [23], we determined that each condensin gene is frequently altered, with shallow and deep deletions occurring in many cancer types. Furthermore, in CRC patients we show that reduced expression (mRNA) of all eight genes is associated with worse overall survival. Next, we employed our established single cell quantitative imaging microscopy (scQuantIM) approaches [24] to accurately assess CIN by evaluating the cell-to-cell heterogeneity in genetic and/or chromosomal changes that arise in a given population [4,5]. Using HCT116, a karyotypically stable cell line, we show that reduced expression corresponds with increases in cell-to-cell heterogeneity and significant changes in key phenotypes associated with CIN, including nuclear areas (NAs), micronucleus (MN) (extranuclear bodies found outside the primary nucleus, see [25]) formation, and chromosome complements. Finally, by extending our study into a second karyotypically stable cell line (hTERT), we show the impact that reduced condensin gene expression has on CIN is independent of cell type. Accordingly, our collective findings show that reduced condensin gene expression underlies CIN, which along with the TCGA data, strongly implicate reduced condensin gene expression as a pathogenic driver of CRC, with implications for oncogenesis in many additional cancer types.

## 2. Results

### 2.1. Condensin Genes Are Frequently Altered in Cancer and Correlate with Worse Patient Outcomes in Colorectal Cancer

**Figure 1:**
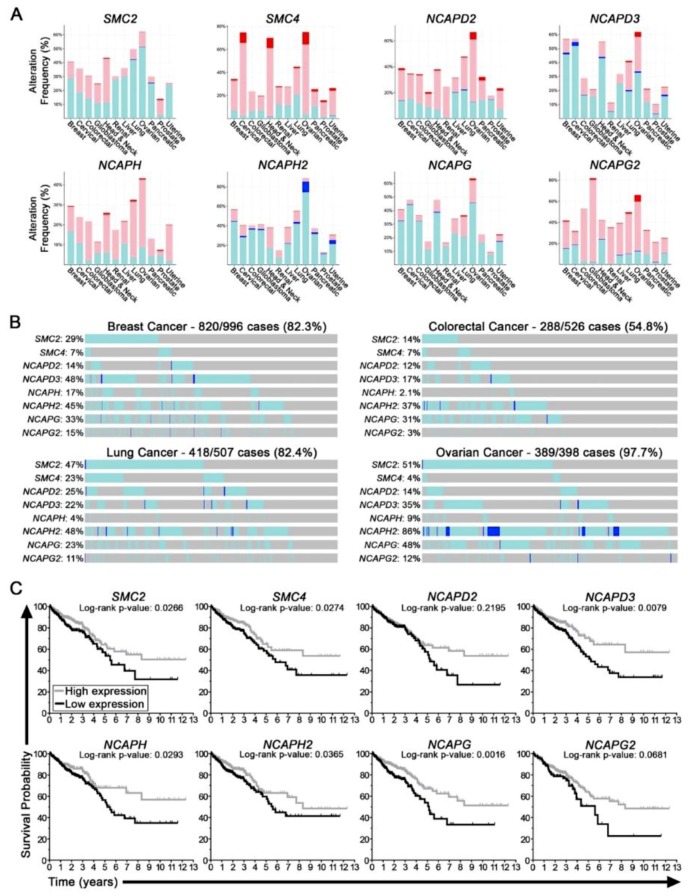
Condensin gene alterations correspond with reduced expression and worse patient survival in cancer. (**A**) Frequency of condensin gene copy number alterations in 12 common cancer types [23]; shallow deletions (aqua), deep deletions (blue), small-scale gains (pink) and large-scale amplifications (red). (**B**) The cumulative frequency of shallow and deep deletions for all condensin genes in breast (82.3%), colorectal (54.8%), lung (82.4%), and ovarian (97.7%) cancers [23]. (**C**) Kaplan–Meier curves reveal that CRCs with reduced condensin gene expression (mRNA) typically correlate with worse overall survival relative to those with high expression [23]. Log-rank tests identify significantly worse outcomes for all genes, with the exception of *NCAPD2* (*p* = 0.2195) and *NCAPG2* (*p* = 0.0681), which do show similar trends.

Using publicly available patient derived datasets obtained from TCGA [23], we determined that all eight condensin genes (*SMC2, SMC4, NCAPD2, NCAPD3, NCAPG, NCAPG2, NCAPH,* and *NCAPH2*) are frequently altered in at least 12 common cancer types (Figure 1A). Moreover, in all 12 cancers examined, gene copy number alterations are prevalent and include both shallow (≈ heterozygous) or deep (≈ homozygous) deletions that are presumed to be associated with reduced expression. In fact, the cumulative frequency of shallow and deep deletions of all eight genes is substantial, ranging from ~55% in CRC to ~98% in ovarian cancer (Figure 1B). Collectively, these data implicate somatic alterations underlying hypomorphic expression and function of condensin genes as potentially significant etiological events in the development and/or progression of many cancer types. Further support for this possibility comes from the observation that reduced mRNA expression is often associated with worse patient outcomes. In CRC for example, the general trend is that reduced expression of all eight genes is associated with worse overall patient survival (Figure 1C). In fact, reduced expression was deemed statistically significant for all condensin genes, with the exception of *NCAPD2* (*p* = 0.22) and *NCAPG2* (*p* = 0.07), which are not significant but do exhibit similar trends. Collectively, these findings identify reduced condensin gene expression as a potential pathogenic event underlying the development and/or progression of many cancer types, thus, warranting further molecular study.

### 2.2. Diminished Condensin Gene Expression Induces Increases in Nuclear Areas (NAs) and Micronucleus (MN) Formation in HCT116 Cells

**Figure 2:**
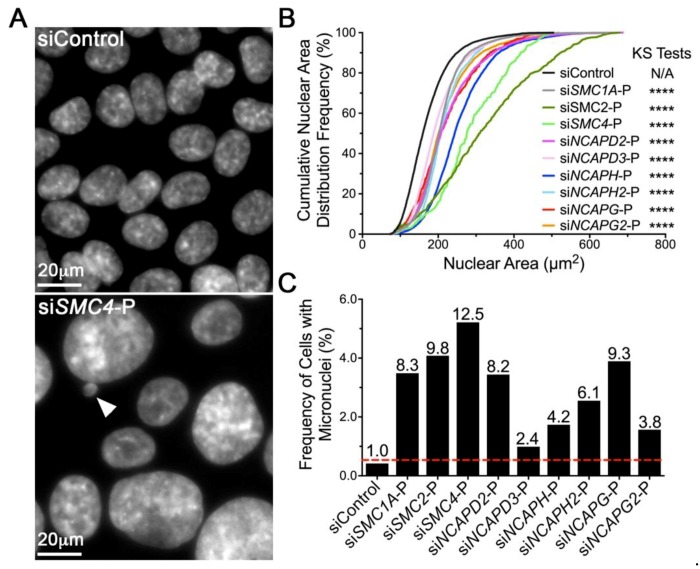
Reduced condensin gene expression induces significant changes in NAs and MN formation in HCT116. (**A**) Representative micrographs depicting visual differences in NAs and MN formation (arrowhead) following condensin gene silencing using siRNA pools (-P) relative to siControl. Note the scale bars are identical. (**B**) Cumulative NA distribution frequencies following condensin gene silencing relative to siControl (black). Kolmogorov-Smirnov (KS) tests reveal statistically significant increases in NA distributions (rightward shift) following silencing relative to siControl (N/A, not applicable; ****, *p* < 0.0001). (**C**) Column graph presenting the mean frequency of cells with micronuclei following silencing, with the mean fold increase (relative to siControl) indicated above each column. The red dashed line identifies the minimum threshold (mean + 2 standard deviations of siControl) required to be considered a significant increase in MN formation.

To determine the impact diminished expression has on CIN, a comprehensive siRNA-based screen was performed in which all eight condensin genes were independently assessed. More specifically, scQuantIM was employed to identify changes in two CIN associated phenotypes, NAs and MN formation [24]. Conceptually, changes in NAs are typically associated with large-scale changes in DNA content, while MN formation is often associated with small-scale changes in DNA content arising from chromosome missegregation events [26,27]. Importantly, all work was performed in HCT116, a karyotypically stable CRC cell line (modal number of 45 chromosomes) that has been used extensively in similar CIN based studies [24,28,29,30]. Overall, gene silencing was associated with visual increases in NAs (Figure 2A) that correspond with increases in NA heterogeneity, mean NAs (Table S1) and significant increases (*p* < 0.0001) in cumulative NA distribution frequencies relative to the control (Figure 2B). In addition, reduced expression corresponded with 2.4- to 12.5-fold increases in MN formation relative to the control (Figure 2C; Table S2). Interestingly, *SMC2* and *SMC4* silencing induced the largest increases in cumulative NA distributions and MN formation, which likely reflects their central roles with both condensin complexes. Collectively, the data gleaned from this screen provide initial mechanistic insight of how reduced condensin gene expression may underlie CIN and contribute to oncogenesis.

### 2.3. Reduced SMC2 Expression Is Associated with CIN and Chromosome Decompaction

**Figure 3:**
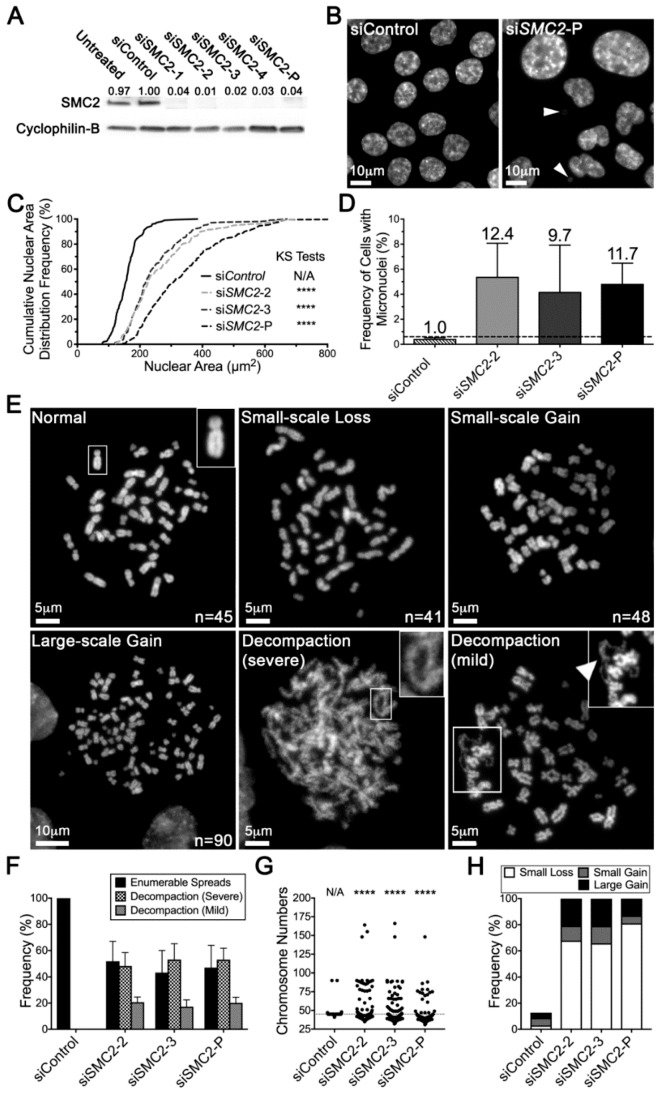
*SMC2* silencing induces CIN phenotypes in HCT116 cells. (**A**) Western blot showing SMC2 levels following silencing with either individual (si*SMC2*-1, -2, -3, or -4) or pooled (si*SMC2*-P) siRNA duplexes; Cyclophilin B is the loading control. Semi-quantitative image analysis was performed where SMC2 levels were first normalized to the respective loading control and are presented relative to siControl (1.00). See Figure S1 for detailed information. (**B**) Representative images depicting visual increases in NA heterogeneity and MN formation (arrowheads) following *SMC2* silencing relative to siControl. (**C**) Cumulative NA distribution graph for *SMC2* silenced conditions relative to siControl. KS tests reveal statistically significant increases in cumulative NA distribution frequencies following *SMC2* silencing (siSMC2-2, -3, and -P) relative to siControl (N/A, not applicable; ****, *p* < 0.0001). (**D**) Column graph presenting the mean MN formation + standard deviation (SD) following *SMC2* silencing; fold increase relative to siControl is presented above each column. The horizontal line indicates the minimum threshold (mean + 2 SD of siControl) required to be considered a significant increase in MN formation relative to siControl. (**E**) Representative images of various phenotypes observed in mitotic chromosome spreads including, normal (top left, inset presents a single magnified normal chromosome), small-scale chromosome losses (top middle), small-scale gains (top right) and large-scale gains (bottom left), along with severe (bottom middle, inset presents a magnified portion of a decondensed chromosome) and mild (bottom right) chromosome decompaction phenotypes. Chromosome numbers are presented in the bottom right corner of those spreads that could be accurately enumerated. (**F**) Bar graph showing the frequency of enumerable mitotic chromosome spreads (+ SD) versus those with severe or mild chromosome decompaction phenotypes. Note that a subset of the mild, but none of the severe decompaction phenotypes, could be accurately enumerated. (**G**) Dot plot presenting the individual chromosome numbers for each enumerable mitotic chromosome spread. Horizontal line identifies the modal chromosome number (45) for HCT116. KS-tests reveal statistically significant changes in the cumulative frequency distributions of chromosome numbers following *SMC2* silencing relative to siControl (****, *p* < 0.0001). (**H**) Bar graph showing overall increases in the frequency of aberrant chromosome numbers following *SMC2* silencing relative to siControl, which includes small-scale losses, small-scale gains, and large-scale gains.

To confirm that the above changes in NA and MN formation were not due to off-target effects, direct tests were performed with individual and pooled siRNAs. *SMC2* was purposefully selected for initial validation as it induced strong CIN phenotypes in the initial screen. We first established the silencing efficiencies of the individual and pooled siRNAs to ultimately identify the two most efficient individual duplexes. As shown in Figure 3A, SMC2 levels were typically reduced to 11% or less of endogenous levels (siControl), with si*SMC2*-2 (4% of endogenous levels) and si*SMC2*-3 (5%) identified as the two most efficient individual duplexes. These duplexes, along with the si*SMC2*-Pool (11%) were employed in all subsequent work aimed at evaluating the impact that reduced expression has on NAs and MN formation. As with the initial screen, reduced *SMC2* expression was associated with visual increases in NAs (Figure 3B) that corresponded with statistically significant increases in cumulative NA distribution frequencies (Figure 3C, Table S3). Reduced expression also induced large increases in MN formation, specifically 12.4-, 9.7-, and 11.7-fold for si*SMC2*-2, si*SMC2*-3, and si*SMC2*-P, respectively (Figure 3D, Table S4).

To firmly establish *SMC2* as a novel CIN gene, we sought to determine whether the increases in NAs and MN formation corresponded with alterations in chromosome numbers (i.e., N-CIN). To formally test this possibility, mitotic chromosome spreads were generated from the *SMC2* silenced and control cells, and chromosomes were manually enumerated from a minimum of 100 spreads/condition. Overall, reduced *SMC2* expression was generally associated with two highly reproducible and distinct phenotypes (Figure 3E): (1) aberrant chromosome numbers including small-scale losses, small-scale gains, and large-scale gains; and (2) defective mitotic figures [25] displaying a severe chromosome decompaction/decondensation phenotype involving all chromosomes, or a mild decompaction/decondensation phenotype involving a subset of chromosomes. Overall, *SMC2* silencing corresponded with a high frequency of aberrant spreads, with ~96%–99% exhibiting either numerical and/or decompaction phenotypes. Furthermore, and in agreement with a central role in mitotic chromosome condensation, ~48%–53% of *SMC2* silenced spreads exhibited severe decompaction phenotypes, while ~17%–21% exhibited mild phenotypes; and no decompaction phenotypes were observed within the siControl spreads (Figure 3F). Although the severe decompaction phenotype prevented us from enumerating chromosomes from a subset of spreads, we did identify a large increase in cell-to-cell heterogeneity (Figure 3G) and significant changes in the cumulative distribution frequencies of chromosome numbers relative to the controls within the remaining subset (Table S5). More specifically, ~28%–38% exhibited small-scale chromosome losses, ~2%–6% exhibited small-scale chromosome gains, and ~6%–11% harbored large-scale chromosome gains as compared to ~3%, ~6%, and ~4%, respectively, within the siControl (Figure 3H). Collectively, these data identify *SMC2* is a novel CIN gene and, furthermore, show that reduced *SMC2* expression induces chromosome decompaction phenotypes, which may be a significant driver of the abnormal chromosome numbers observed within these cells.

### 2.4. Condensin Gene Silencing Induces Numerical CIN and Structural Defects in HCT116 Cells

**Figure 4:**
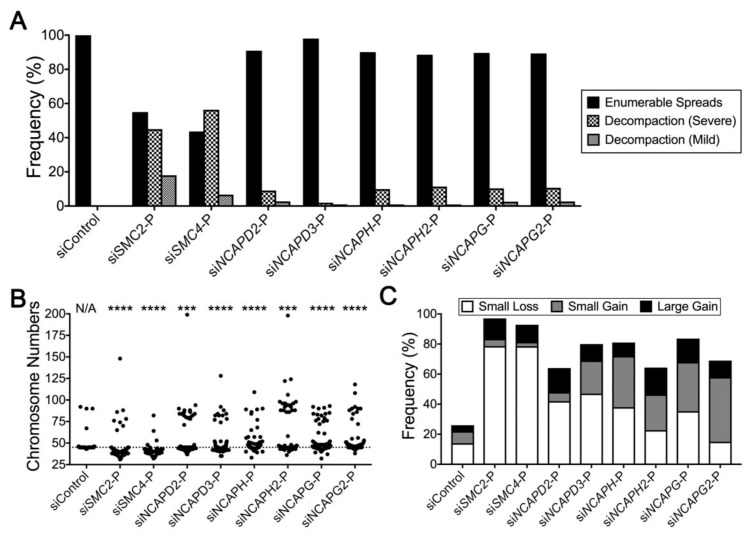
Reduced condensin gene expression induces aberrant numerical and decompaction phenotypes in HCT116. (**A**) Bar graph presenting the frequency of enumerable and chromosome decompaction phenotypes (severe and mild) following gene silencing. (**B**) Dot plot depicting an overall increase in chromosome number heterogeneity following condensin gene silencing relative to siControl. The horizontal line identifies the modal chromosome number of HCT116. KS tests reveal statistically significant differences in the cumulative distribution frequencies of chromosome numbers relative to siControl (***, *p* < 0.001; ****, *p* < 0.0001). (**C**) Bar graph depicting overall increases in the frequency of aberrant chromosome numbers following condensin gene silencing.

Having originally identified changes in NAs and MN formation for the seven remaining condensin genes (Figure 2), we now sought to determine whether their reduced expression also induced changes in chromosome numbers (i.e., numerical CIN) and/or decompaction phenotypes. Western blot analyses were performed and confirmed efficient gene silencing in HCT116 cells (Figure S3). Briefly, each gene was silenced, mitotic chromosome spreads were generated, and a minimum of 100 spreads/condition were imaged and analyzed, as above. In general, gene silencing corresponded with overall increases in severe and mild decompaction phenotypes and changes in chromosome numbers relative to the control (Figure 4A), with the most pronounced phenotypes occurring within the *SMC4* silenced cells. Interestingly, the *SMC4* results (56% severe, 7% mild) appear most similar to those of *SMC2* (45% severe, 18% mild), with the remaining six genes exhibiting similar frequencies of severe (~2%–11%) and mild (~1%–7%) decompaction phenotypes. Within the enumerable spreads, gene silencing also corresponded with increased heterogeneity in chromosome number (Figure 4B) that coincided with significant differences in cumulative distribution frequencies relative to the control (Table S6). Furthermore, following gene silencing, Figure 4C shows there are overall increases in the frequency of mitotic chromosome spreads with aberrant chromosome numbers for all condensin genes (64%–97%) relative to the control (26%). In general, this includes increases in small-scale losses and large-scale chromosome gains for all genes evaluated. Interestingly, increases in small-scale chromosome gains also occur, but tend to be restricted to the five genes (*NCAPD3*, *NCAPH*, *NCAPH2*, *NCAPG*, *NCAPG2*) that encode proteins that are not shared between the two condensin complexes. Collectively, the above data further support the condensin complex and each individual member as a CIN gene in HCT116 cells. The findings also highlight key roles of SMC2 and SMC4 in chromosome condensation and CIN, which likely stems from their critical involvement in both condensin I and II complexes. In addition, they also suggest potential functional redundancy exists between the remaining complex members as they belong to either, but not both, condensin complexes.

### 2.5. Reduced Condensin Gene Expression Induces CIN Phenotypes That Are Independent of Cell Type

**Figure 5:**
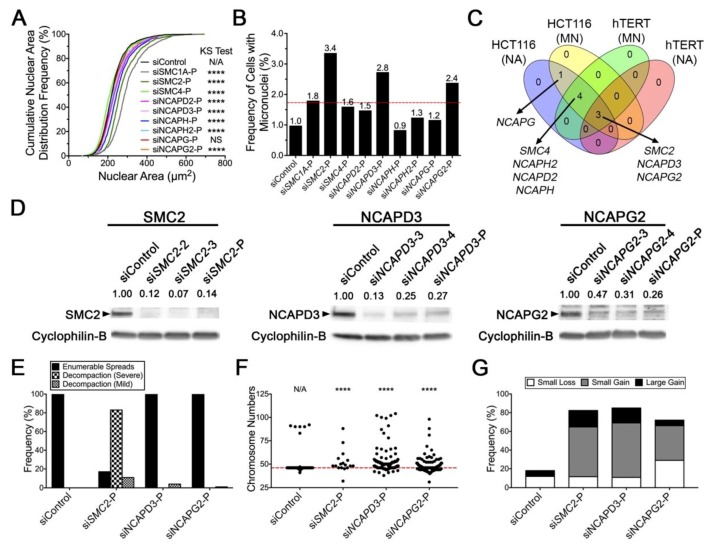
Condensin gene silencing induces increases in CIN phenotypes in hTERT cells. (**A**) Cumulative NA distribution frequencies following condensin gene silencing in hTERT cells relative to siControl (black). KS tests reveal reduced expression induces statistically significant changes in NA distributions, with the exception of *NCAPG* (*p* = 0.07) (N/A, not applicable; N/S, not significant (*p* > 0.01); ****, *p* < 0.0001). (**B**) Column graph presenting the frequency of MN formation following silencing. The fold increase in mean MN formation relative to siControl is presented above each column, while the horizontal line identifies the minimum threshold to be considered a significant increase. (**C**) Venn diagram presenting the results of the NA and MN formation assays performed in both HCT116 and hTERT cells. Note that *SMC2*, *NCAPD3,* and *NCAPG2* silencing induced significant phenotypes in all four assay/cellular contexts. (**D**) Representative Western blots showing SMC2 (left), NCAPD3 (middle) and NCAPG2 (right) levels following silencing in hTERT cells with both individual and pooled siRNAs; Cyclophilin B is the loading control. Semi-quantitative image analysis was performed, and residual protein levels are presented relative to siControl (1.00). See Figure S2 for detailed information. (**E**) Bar graph showing the frequency of enumerable mitotic spreads versus those with severe or mild chromosome decompaction phenotypes. Note that the majority of *SMC2* silenced cells present with decompaction phenotypes, including ~83% and ~11% with severe and mild phenotypes, respectively. (**F**) Dot plot presenting the individual chromosome numbers for each enumerable mitotic chromosome spread, with the horizontal line identifying the modal chromosome number (46) of hTERT cells. Note that only 17% of spreads generated following *SMC2* silencing could be enumerated due to the prevalent severe decompaction phenotype. KS tests reveal significant differences in the distribution of chromosome numbers relative to siControl (N/A, not applicable; ****, *p* < 0.0001). (**G**) Bar graph depicting increases in the overall frequency of aberrant chromosome numbers relative to siControl, which includes small-scale losses, small-scale gains and large-scale gains. Note that only 17/100 mitotic chromosome spreads could be enumerated following *SMC2* silencing, 14 of which, exhibited numerical deviations from the modal number.

To determine whether our findings are independent of cell type, an analogous series of experiments were performed in a second karyotypically-stable cell line. The hTERT cells were purposefully selected as they are a non-malignant, human telomerase immortalized cell line with a modal number of 46 chromosomes that have been used extensively in similar CIN-based studies [24,29,30]. Overall, gene silencing (Figure S4) was typically associated with increases in NAs and MN formation, albeit to lesser extent than that observed in HCT116 cells. For example, gene silencing induced significant differences in cumulative NA distribution frequencies (Figure 5A), with the exception of NCAPG (*p* = 0.07, Table S7). Although MN formation was typically increased for most genes (Table S8), it only reached the minimum threshold value for three genes, *SMC2* (3.4-fold), *NCAPD3* (2.8-fold), and *NCAPG2* (2.4-fold) (Figure 5B). To identify candidates to pursue in subsequent validation studies, the NA and MN formation data from HCT116 and hTERT were assessed (Figure 5C), and *SMC2*, *NCAPD3,* and *NCAPG2* were selected as the strongest candidates as they induced significant differences in both assays in both cell lines. Next, Western blots were used to determine the silencing efficiencies of the two most efficient silencing duplexes along with the pool (Figure 5D). Although all silencing conditions corresponded with reduced protein expression, they were generally not reduced to the same extent as in HCT116 cells. In agreement with the HCT116 data, reduced expression coincided with increases in aberrant phenotypes within the mitotic chromosome spreads (Figure 5E). More specifically, *SMC2* silencing induced severe and mild decompaction phenotypes in 83% and 11% of spreads, respectively, while *NCAPD3* and *NCAPG2* silencing did not produce any severe decompaction phenotypes, but rather, generated mild decompaction phenotypes in 4% and 1% of spreads, respectively. Gene silencing also corresponded with an overall increase in heterogeneity and cumulative frequency distributions of chromosome numbers within the enumerable spreads (Figure 5F) that are statistically significant (Table S9). In fact, ~72%–85% of spreads exhibited deviations from the modal (46) number of chromosomes (Figure 5G) relative to 18% for siControl, with increases in small scale chromosome gains being the most prevalent. However, increases in small scale losses were also observed following *NCAPG2* silencing, while increases in large scale gains occurred following *SMC2* and *NCAPD3* silencing. Collectively, the above findings show that condensin gene silencing is associated with increases in NA and MN formation, and that subsequent direct tests validate *SMC2*, *NCAPD3,* and *NCAPG2* as novel CIN genes in hTERT cells, and establish that their impacts are independent of cell type.

## 3. Discussion

In this study, we sought to comprehensively assess the impact that reduced expression of eight key condensin genes has in cancer. Using publicly available datasets, we show that all eight genes are somatically altered in numerous cancer types, and that deletions (shallow and deep) collectively occur in 50%–90% of common cancer types. Furthermore, reduced expression of each gene coincides with worse patient survival in CRC, suggesting reduced expression may have pathogenic implications for cancer. To explore this possibility, an initial screen coupling reverse genetics and scQuantIM was employed to systematically assess each gene in HCT116. In agreement with a central role in genome stability, reduced expression induced increases in NA and MN formation along with increases in chromosome decompaction phenotypes and changes in chromosome numbers. Finally, we extended our study into hTERT cells and obtained similar, albeit less pronounced phenotypes, demonstrating that reduced condensin gene expression induces CIN independently of cell type. In summary, our findings show that, collectively, reduced condensin gene expression is prevalent in cancer, is associated with worse patient outcomes, and underlies increases in CIN. Thus, our clinical and fundamental findings suggest that somatic mutations adversely impact the expression and/or encoded function of any condensin gene and may be a significant pathogenic driver contributing to the development and progression of many cancers, including CRC.

In keeping with their conserved roles in both condensin I and II complexes, *SMC2* and *SMC4* silencing induced the strongest decompaction phenotypes and numerical changes, while silencing the six remaining genes was typically associated with milder decompaction phenotypes and fewer numerical alterations. In fact, severe decompaction phenotypes were approximately five to eight times more abundant following *SMC2* (45%) and *SMC4* (56%) silencing than silencing the remaining six genes (2%–11%) in HCT116 cells. The milder phenotypes associated with *NCAPD2*, *NCAPD3*, *NCAPH*, *NCAPH2*, *NCAPG,* or *NCAPG2* silencing, may simply reflect their unique roles within only one of the complexes and the potential for functional redundancy existing between the two complexes, particularly as it pertains to mitotic chromosome compaction [16,17]. It was also noted that the aberrant phenotypes were typically more pronounced within HCT116 cells, which is in agreement with other studies involving both cell lines [24,29,30,31]. Although the underlying biological mechanism(s) accounting for this difference is unclear, it may simply reflect the enhanced silencing observed within HCT116 relative to hTERT. Alternatively, the differences may also be due to inherent differences existing between the two cell lines. For example, HCT116 is a transformed CRC cell line with an established DNA mismatch repair deficiency stemming from a *MLH1* defect [32], while hTERT is a non-malignant, immortalized (human telomerase reverse transcriptase) fibroblast cell line that is DNA repair proficient [33]. By definition, a *MLH1* deficiency enables the accrual of additional somatic mutations over time that may genetically synergize with reduced condensin gene expression to induce more robust CIN phenotypes. In contrast, others have shown that re-expression of *TERT* confers increased genome stability and enhances DNA repair, which may render hTERT cells less prone to the development of CIN phenotypes [34]. Beyond these genetic susceptibilities, HCT116 (~22 h) and hTERT (~36 h) cells have different doubling times, which over the four day time course of these experiments, equates to on average approximately 4.4 and 2.7 population doublings, respectively. Thus, the additional population doublings may provide HCT116 cells additional opportunities for CIN to occur (e.g., additional cycles through mitosis may result in more chromosomal segregation errors). Accordingly, future studies are essential to determine the impact that reduced condensin gene expression has on CIN in both early disease models with little to no background mutations, and late disease models harboring extensive mutations.

Our findings that reduced condensin gene expression induces CIN have tremendous implications for cancer and are in agreement with similar data obtained in model organisms. For example, others have shown that reduced expression or mutant expression of *Smc4* in yeast induce chromosome transmission fidelity defects [22], while conditional knockout of mouse *Smc2* and *Ncaph2* are associated with increases in aberrant chromosome morphology and chromosome missegregation events, that were not observed following conditional knockout of *Ncaph* [35]. Furthermore, Martin et al. [21] showed that reduced expression and specific mutations in *NCAPD2*, *NCAPH,* and *NCAPD3* cause microcephaly through mitotic chromosome segregation errors. Indeed, we show that copy number losses (i.e., shallow and deep deletions) of condensin genes occur frequently in many cancers, but remarkably are collectively altered in ~98% of all ovarian cancers. This high frequency of copy number losses suggests condensin genes may be selectively targeted and drive the development and progression of ovarian cancer, although the aberrant mechanism(s) underlying these copy number losses remains to be determined. Our molecular investigations and insight may also help interpret the associations identified by others. For example, Kar et al. [36] employed pairwise meta-analyses to identify shared associations between breast and ovarian cancer risk. They identified an index variant (rs200182588) at the *SMC2* locus (9q31) that localizes to the 5′-untranslated region of *SMC2*. On the basis of our clinical and mechanistic findings, it is tempting to speculate that this alteration may adversely impact mRNA stability and/or expression resulting in reduced condensin function that induces CIN and contributes to breast and ovarian cancer pathogenesis. Condensin is also emerging as a key regulator in the DNA damage response (reviewed in [37]). While condensin I harbors a role in DNA single-strand break repair by interacting with PARP1-XRCC1 complex [38,39], it does not appear to have a significant role in DNA double-strand break repair. In contrast, condensin II functions in double-strand break repair. Moreover, condensin II depletion only impacts homology directed repair of double-strand breaks induced with ionizing radiation, but is dispensable for G2/M checkpoint activation [40]. Furthermore, genotoxic stress induces DNA double-strand breaks that, if not repaired correctly, can result in acentric chromosome fragments. Due to the lack of a centromere, these fragments are incapable of undergoing proper chromosome segregation during mitosis and can result in the formation of micronuclei. Interestingly, our study shows that reduced expression of both condensin I and II members induces increases in MN formation, suggesting reduced expression and/or function of either complex underlies increases in DNA double-strand breaks. Although the mechanism(s) accounting for MN formation are not immediately evident, several possibilities exist to explain how reduced expression of condensin I and/or condensin II complex members may have a role. First, it is conceivable that during S-phase, replication stress underlying single-strand breaks could induce replication fork collapse and the formation of double-strand breaks resulting in acentric chromosome fragments unable to undergo proper chromosome segregation leading to MN formation [41,42]. Second, as condensin I does not appear to function in DSB repair, it is plausible that the micronuclei arise due to the missegregation of whole chromosomes, rather than acentric fragments. Finally, studies in *Saccharomyces cerevisiae* [43] and *Caenorhabditis elegans* [44] show condensin is essential for axial compaction of pericentric chromatin. Thus, it remains possible that reduced condensin gene expression/function could negatively impact the architecture of the pericentric chromatin to adversely affect kinetochore formation and/or microtubule attachments to prevent proper chromatid segregation. Accordingly, future studies are required to explore the individual contributions condensin I and II members may have in these and other mechanisms underlying MN formation.

Recent data show that CIN is associated with cellular transformation, intratumoral heterogeneity, aggressive tumors, metastasis, and poor patient prognosis. However, more recent data suggest targeting the molecular determinants of CIN may hold tremendous potential for precision medicine in two specific ways (reviewed in [45]). First, synthetic lethal approaches could be devised to target the aberrant genes giving rise to the CIN phenotype. Synthetic lethality refers to the lethal combination of two independently viable mutations, which in a cancer context seeks to exploit somatic alterations in key driver genes, including CIN genes. In this regard, synthetic lethal strategies could be developed that elicit a highly specific killing of cancer cells harboring somatic losses in condensin genes. The second approach builds on the paradoxical observation that in some cancers (e.g., breast), high levels of CIN are correlated with better patient outcomes [46]. The basis for this paradoxical observation is not initially clear, but it may be due to the inability of a cell to manage and cope with extreme gains and/or losses of chromosomes. Indeed, it has been proposed that extreme levels of CIN are not compatible with viability [46] (reviewed in [45,47]). Thus, the CIN that is induced following reduced condensin gene expression could be further exacerbated above a critical threshold required to induce death by targeting other known CIN genes. Alternatively, perhaps leveraging the CIN already present within many cancers by selectively targeting key condensin genes may be sufficient to cause the extreme levels of CIN required to induce highly selective killing. In any case, understanding the contribution that reduced condensin gene expression has on patient outcomes, CIN, and cancer pathogenesis will have tremendous implications for future precision medicine strategies aimed at improving the lives and outcomes of those living with cancer.

## 4. Materials and Methods

### 4.1. The Cancer Genome Atlas—Gene Alteration and Outcome Analyses

Publicly available genomic and mRNA expression data were freely obtained from TCGA (https://portal.gdc.cancer.gov/) [23]. Genomic data were extracted from 12 common cancer types (breast, cervical, colorectal, glioblastoma, head and neck, renal, liver, lung, ovarian, pancreatic, prostate, and uterine) using web-based analysis and visualization tools (cBioPortal; www.cbioportal.org) with user-defined onco-query commands, including HETLOSS, HOMDEL, AMP, and GAIN [48]. The raw mRNA expression data and clinical outcomes were exported from TCGA Research Network and processed in Prism v7 (GraphPad, San Diego, CA, USA). The threshold between high and low mRNA expression was determined as described elsewhere [49]. Briefly, for each gene, the threshold was selected as the mRNA expression level between the 20th and 80th percentile that results in the lowest log-rank *p*-value in the survival analysis comparing patients with high or low mRNA expression. The Kaplan–Meier curves were generated on the basis of this analysis and statistical analyses (log-rank tests) were performed (*p*-value < 0.05 is significant). All figures were assembled in Photoshop CS6 (Adobe, San Jose, CA, USA).

### 4.2. Cell Culture

Two karyotypically stable cell lines were employed throughout this study [29]. HCT116 (human colorectal carcinoma) were purchased from the American Type Culture Collection (Rockville, MD, USA), while hTERT (human telomerase immortalized skin fibroblasts) were generously provided by Dr. C.P. Case (University of Bristol, Bristol, UK). Cells were maintained in McCoy’s 5A (HCT116) or Dulbecco’s modified Eagle’s media (hTERT), supplemented with 10% fetal bovine serum. Cell lines were authenticated on the basis of growth, morphology, and spectral karyotyping [29]. All cells were maintained at 37 °C in a humidified incubator containing 5% CO_2_.

### 4.3. Gene Silencing and Western Blot Analysis

ON-TARGET*plus* (Dharmacon) siRNAs targeting each of the eight condensin genes (*SMC2, SMC4, NCAPD2, NCAPD3, NCAPG, NCAPG2, NCAPH,* and *NCAPH2*), and both positive (si*SMC1A*) [24,30] and negative (*GAPDH*; siControl) [28,30] controls were used either individually (-1, -2, -3 or -4) or as pools (-P) comprised of four distinct siRNAs targeting distinct coding region sequences as detailed previously [29]. In general, cells were seeded, permitted to attach and grow for 24 h prior to being transfected, while gene silencing was assessed 4 days post transfection using standard Western blotting approaches [29] with the antibodies/dilutions indicted in Table 1. Semi-quantitative immunoblot analyses were performed using Image J software as described [29]. Figures were assembled in Photoshop.

### 4.4. Single Cell Quantitative Imaging Microscopy: Nuclear Areas and Micronucleus Formation

ScQuantIM approaches were employed to evaluate changes in NA and MN formation following gene silencing as detailed elsewhere [24]. Briefly, 500 HCT116 or 1000 hTERT cells were seeded into a 96-well plate, permitted to attach (24 h) and transfected with siRNAs as above. Four days post transfection, cells were fixed, counterstained (Hoechst 33342), and imaged (3 × 3 matrix of non-overlapping images/condition) using a Cytation 3 Imaging Multi-Mode Reader (BioTek, Winooski, VT, USA) equipped with a 16 bit CCD camera, a 20x Olympus LUCPLFLN lens (numerical aperture = 0.45) and Gen5 software (BioTek). Gen5 software was used to quantify NAs from a minimum of 200 nuclei/condition using the following criteria: (1) a maximal mean fluorescence signal intensity was used to exclude brightly stained bodies such as mitotic or apoptotic cells; and (2) the ”split touching objects” feature was used to segment the image (i.e., resolve spatially proximal nuclei). To quantify changes in MN formation, the following two filters were applied to all images: (1) a maximal mean fluorescence signal intensity was used to eliminate mitotic or apoptotic cells; and (2) a size inclusion filter was employed to restrict analyses to Hoechst-stained bodies with a diameter of 1–5 µm (i.e., ≤1/3 the size of a typical nucleus) [24]. The total number of micronuclei per condition were enumerated and expressed as a frequency of the total number of nuclei with micronuclei. Increases in MN formation were operationally defined as significant if increases in MN formation were greater than two standard deviations above the mean value of the siControl. All descriptive statistics (mean, standard deviation, etc.), two sample KS tests and graphs were generated in Prism, while figures were assembled in Photoshop.

### 4.5. Mitotic Chromosome Spreads and Chromosome Enumeration

Mitotic chromosome spreads were generated as detailed elsewhere [29]. Briefly, 35,000 HCT116 or 20,000 hTERT cells were seeded in 6-well plates (24 h), silenced and grown for 4 additional days. Following gene silencing, mitotic chromosome spreads were generated and a minimum of 100 spreads/condition were imaged. Due to the impact on chromosome compaction, spreads were initially scrutinized and classified as either: (1) non-enumerable, in which mild or severe chromosome decompaction events prevented accurate enumeration; or (2) enumerable, where distinct chromosomes could be manually counted. Spreads with numerical differences from the established modal chromosome numbers for HCT116 (45) and hTERT (46) were subsequently classified into one of three categories: (1) small-scale losses involving fewer than 15 chromosomes; (2) small-scale gains involving fewer than 15 chromosomes; and (3) large-scale gains involving more than 15 chromosomes. All data were imported into Prism where descriptive statistics, KS tests, and graphs were generated; figures were assembled in Photoshop.

## 5. Conclusions

In summary, data from this study show that condensin gene copy number variations, particularly deep and shallow deletions, occur frequently in many cancer types, but are collectively observed in approximately 55% of all CRCs. Further, the observation that reduced expression correlates with worse patient outcomes coupled with the molecular data showing reduced expression induces CIN, strongly implicate reduced condensin gene expression as a key pathogenic event in the development and progression of CRC. These findings open novel therapeutic avenues in which the genetic defects underlying reduced condensin gene expression can be leveraged through synthetic lethal strategies to minimize the morbidity and mortality rates associated with CRC.

## Figures and Tables

**Table 1:** List of antibodies employed in Western blot analyses.

Protein Target	Catalogue No.	Source	Host Species	Working Dilution
SMC2	ab10399	Abcam	Rabbit	1:5000
SMC4	ab179831	Abcam	Rabbit	1:1000
NCAPD2	HPA037363	Sigma-Aldrich	Rabbit	1:1000
NCAPD3	ab70349	Abcam	Rabbit	1:1000
NCAPG	SAB1405247	Sigma-Aldrich	Rabbit	1:1000
NCAPG2	ab70350	Abcam	Rabbit	1:2000
NCAPH	HPA002647	Sigma-Aldrich	Rabbit	1:1000
NCAPH2	ab71459	Abcam	Rabbit	1:500
Cyclophilin B	ab16045	Abcam	Rabbit	1:30,000

## Supplementary Materials

The following are available online at https://www.mdpi.com/2072-6694/11/8/1066/s1, Figure S1: Raw data of Western blots from Figure 3, Figure S2: Raw data of Western blots from Figure 5, Figure S3: Establishing the silencing efficiencies of condensin gene silencing in HCT116 cells. Figure S4: Assessing residual protein expression following condensin gene silencing in hTERT cells. Table S1: Condensin gene silencing corresponds with significant increases in the cumulative nuclear area distribution frequencies in HCT116 cells. Table S2: Condensin gene silencing induces increases in the MN formation in HCT116 cells. Table S3: *SMC2* silencing corresponds with significant increases in the cumulative NA distribution frequencies in HCT116. Table S4: *SMC2* silencing corresponds with significant increases in the MN formation in HCT116. Table S5: Condensin gene silencing induces significant changes in cumulative frequency distributions for chromosome numbers in HCT116 cells. Table S6: Condensin gene silencing induces significant changes in cumulative frequency distributions for chromosome numbers in HCT116 cells. Table S7: Condensin gene silencing corresponds with significant increases in the cumulative nuclear area distribution frequencies in hTERT cells. Table S8: Condensin gene silencing induces increases in the MN formation in hTERT cells. Table S9: Condensin gene silencing induces increases in the MN formation in hTERT cells.
