# Supplementary material for: Diminished Condensin Gene Expression Drives Chromosome Instability That May Contribute to Colorectal Cancer Pathogenesis
Source: Cancers (Basel). 2019 Jul 28;11(8):1066. doi: 10.3390/cancers11081066 (PMC6721357; doi:10.3390/cancers11081066)
Supplement: Supplementary file 1 [file cancers-11-01066-s001.pdf]

SUPPLEMENTARY INFORMATION

Supplementary Figures

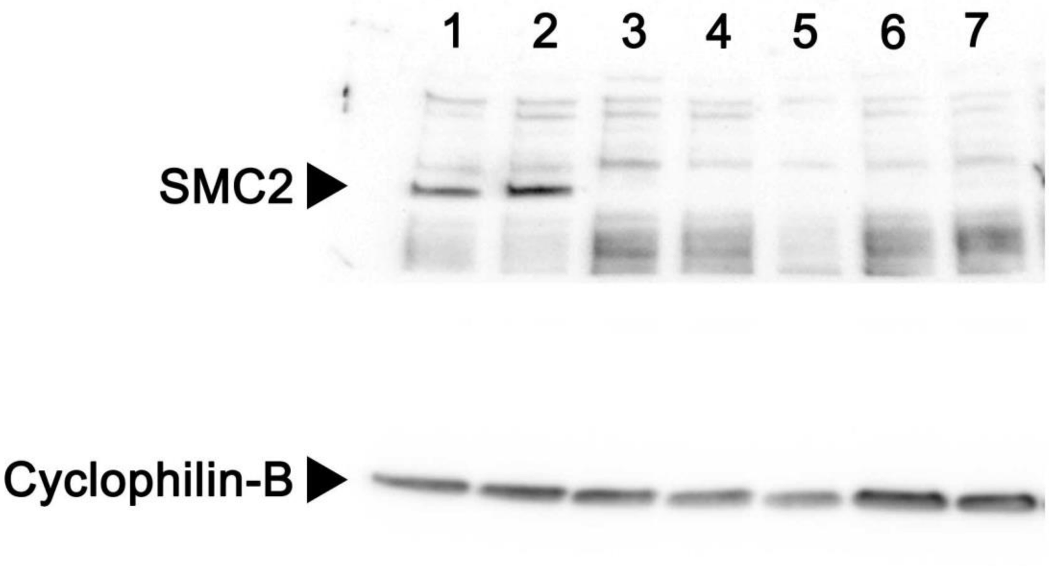

	SMC2	Cyclophilin-B	Ratio SMC2/Cyclo-B	Normalized Ratio
**Lane 1**	39228.472	30035.877	1.306	0.97
**Lane 2**	40285.087	29878.007	1.348	1.00
**Lane 3**	1680.418	28583.392	0.059	0.04
**Lane 4**	630.225	32239.309	0.020	0.01
**Lane 5**	894.205	26993.066	0.033	0.02
**Lane 6**	1983.397	52685.513	0.038	0.03
**Lane 7**	2063.468	34110.551	0.060	0.04

Figure S1: Raw data of western blots from Figure 3.
Western blots of SMC2 (top) and Cyclophilin-B (bottom) in HCT116 cells. Densitometry analyses for SMC2 and Cyclophilin-B were performed using Image J and are indicted. The ratios of SMC2/Cyclophilin-B is shown for each lane, as are the normalized ratios, which are presented relative to the siControl (Lane 2).

A

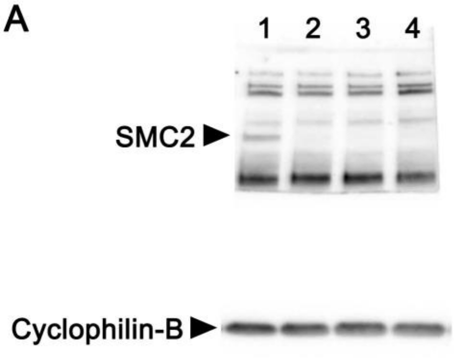

	SMC2	Cyclophilin-B	Ratio S/C	Norm. Ratio
Lane 1	29051.43	24352.95	1.193	1.00
Lane 2	3214.20	22618.36	0.142	0.12
Lane 3	2296.40	27091.41	0.085	0.07
Lane 4	4147.98	24628.46	0.168	0.14

B

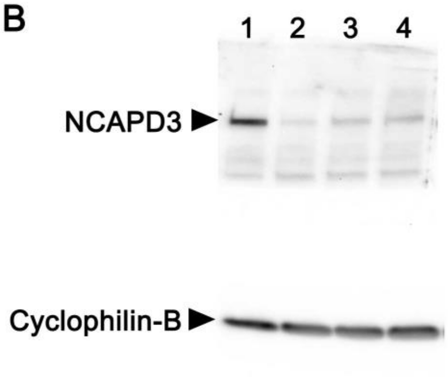

	NCAPD3	Cyclophilin-B	Ratio D3/C	Norm. Ratio
Lane 1	32046.38	21990.53	1.457	1.00
Lane 2	2837.33	17384.10	0.163	0.13
Lane 3	5495.66	18498.61	0.297	0.25
Lane 4	5991.02	26787.02	0.224	0.27

C

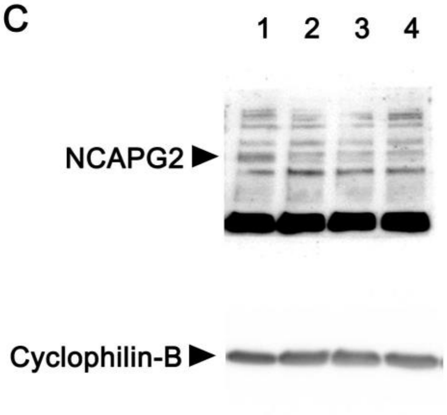

	NCAPG2	Cyclophilin-B	Ratio G2/C	Norm. Ratio
Lane 1	34358.48	17706.90	1.940	1.00
Lane 2	16547.17	18255.94	0.906	0.47
Lane 3	11642.15	19191.74	0.607	0.31
Lane 4	10109.22	19976.17	0.506	0.26

Figure S2: Raw data of western blots from Figure 5.
(**A**) Western blots of SMC2 (top) and Cyclophilin-B (bottom) in hTERT cells. Densitometry measurements are presented for SMC2 and Cyclophilin-B along with the SMC2/Cyclophilin-B ratios and normalized ratios, which are presented relative to the siControl (Lane 1). (**B**) Western blots of NCAPD3 (top) and Cyclophilin-B (bottom) in hTERT cells. Densitometry measurements are presented for NCAPD3 and Cyclophilin-B, while the SMC2/Cyclophilin-B ratios and normalized ratios, which are presented relative to the siControl (Lane 1). (C) Western blots of SMC2 (top) and Cyclophilin-B (bottom) in hTERT cells. Densitometry measurements are presented for SMC2 and Cyclophilin-B along with the SMC2/Cyclophilin-B ratios and normalized ratios, which are presented relative to the siControl (Lane 1).

Figure S3: Establishing the Silencing Efficiencies of Condensin Gene Silencing in HCT116 Cells.

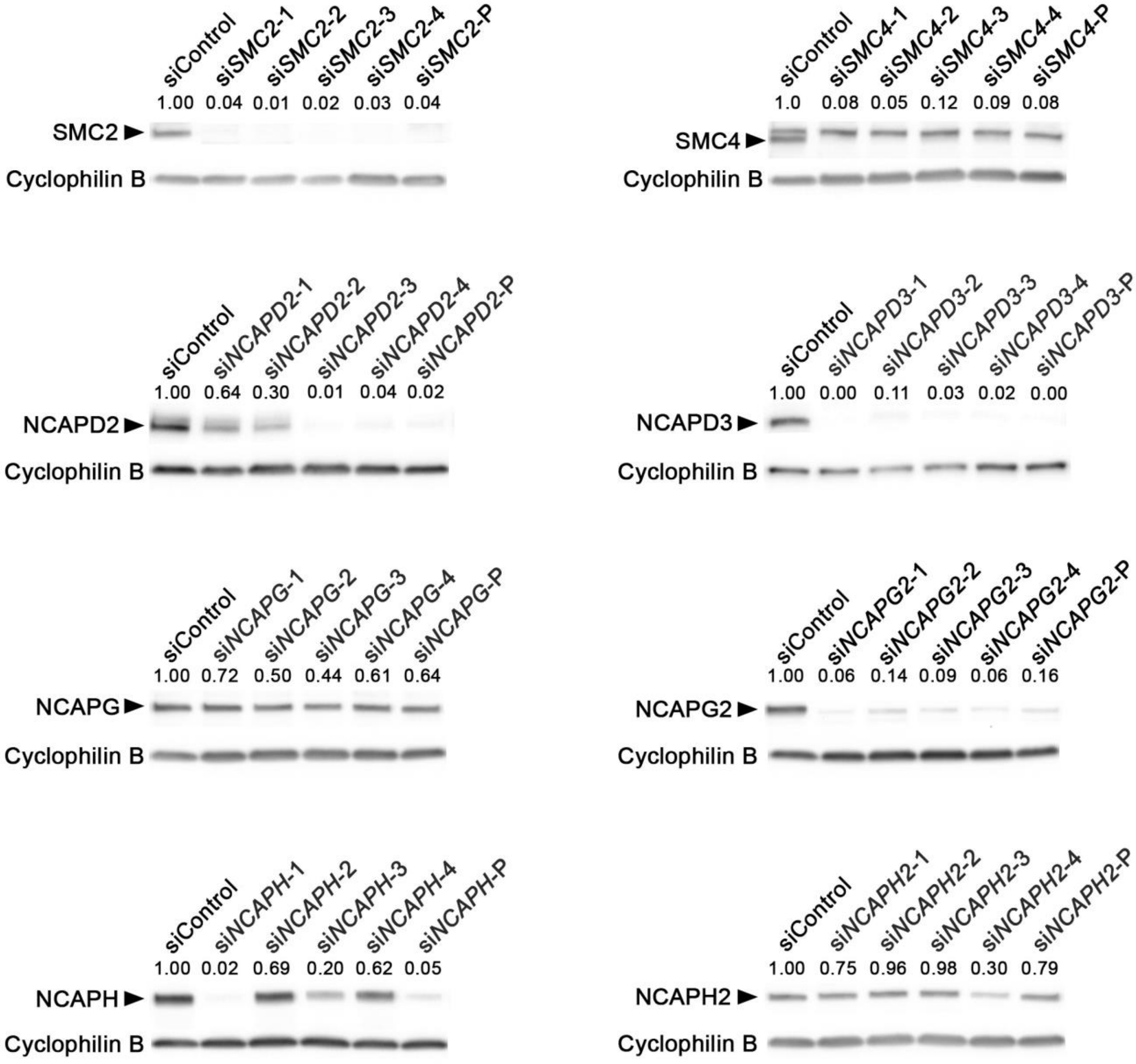

Western blots presenting the residual condensin protein levels (indicated) following silencing with either individual (si*Gene*-1, -2, -3 or -4) or pooled (si*Gene*-P) siRNA duplexes, with Cyclophilin B used as the loading control. Semi-quantitative image analyses were performed whereby residual protein levels were normalized to the respective loading control and are presented relative to the siControl for each gene (1.00).

Figure S4: Assessing Residual Protein Expression Following Condensin Gene Silencing in hTERT Cells.

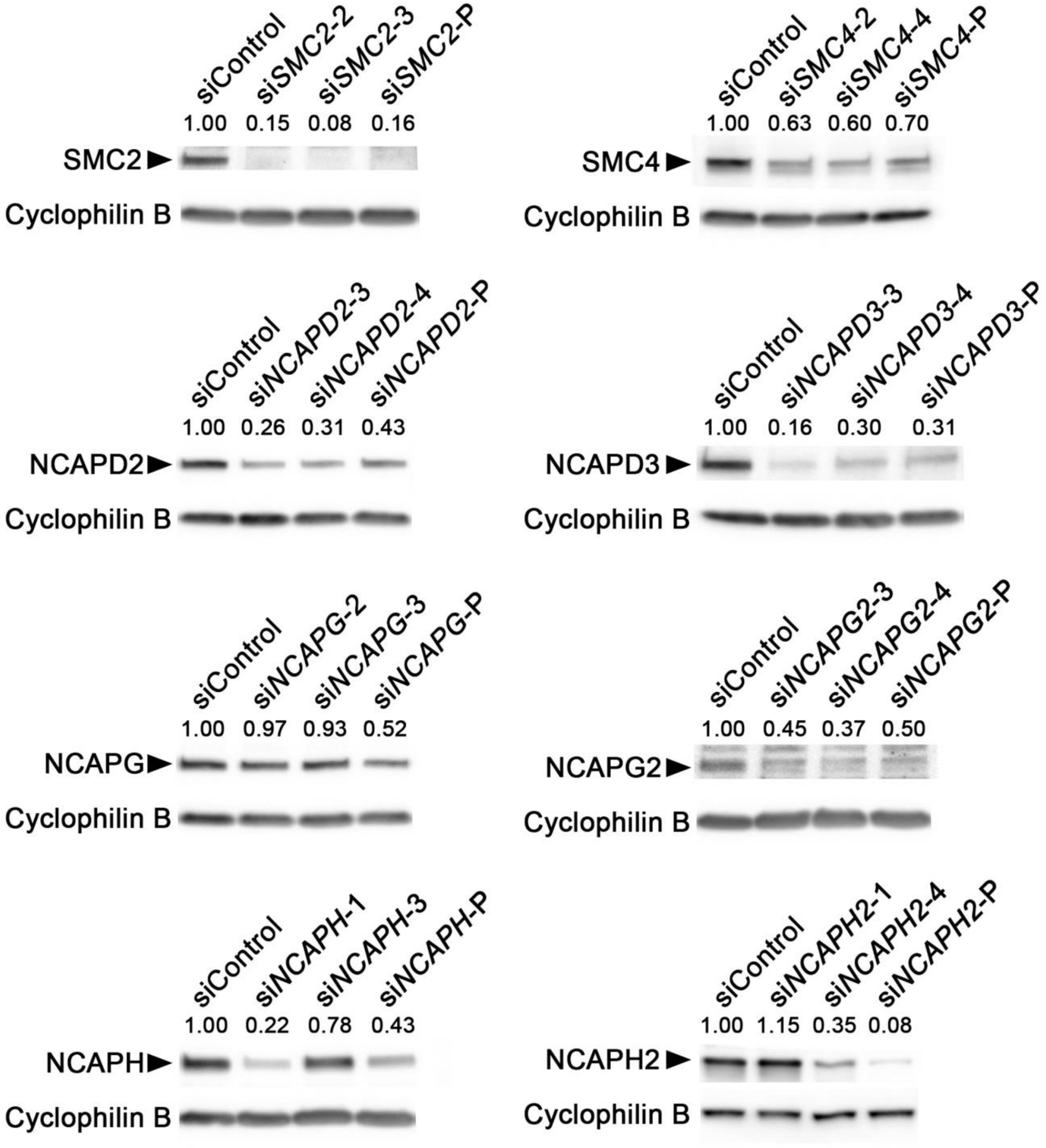

Western blots presenting the residual condensin protein levels (indicated) following silencing with the two most efficient individual (as determined in HCT116; see Supplementary Figure 1), or pooled (si*Gene*-P) siRNA duplexes, with Cyclophilin B used as the loading control. Semi-quantitative image analyses were performed whereby residual protein levels were normalized to the respective loading control and are presented relative to the siControl for each gene (1.00).

Supplementary Tables

Table S1: Condensin Gene Silencing Corresponds with Significant Increases in the Cumulative Nuclear Area Distribution Frequencies in HCT116 Cells.

Cell Line	Condition	N[a]	Mean	Standard Deviation	Fold Increase[b]	CDF *p*[c]
HCT116	siControl	9360	172.6	62.2	N/A	N/A
	si*SMC1A*-P	3602	210.8	64.6	1.22	<0.0001
	si*SMC2*-P	629	325.6	137.8	1.89	<0.0001
	si*SMC4*-P	204	289.9	93.6	1.70	<0.0001
	si*NCAPD2*-P	2381	228.4	86.6	1.32	<0.0001
	si*NCAPD3*-P	5044	202.0	67.0	1.17	<0.0001
	si*NCAPH*-P	3648	257.8	83.6	1.49	<0.0001
	si*NCAPH2*-P	1108	218.6	60.8	1.27	<0.0001
	si*NCAPG*-P	487	224.0	82.5	1.30	<0.0001
	si*NCAPG2*-P	5237	218.6	79.1	1.27	<0.0001

[a]Number (N) of nuclei analyzed.
[b]Fold increase in mean NA relative to siControl (not applicable; N/A).
[c]Two sample KS tests assessing differences in the cumulative distribution frequencies of NAs relative to siControl (N/A, not applicable); $p = <0.01$ is considered statistically significant.

Table S2: Condensin Gene Silencing Induces Increases in the MN Formation in HCT116 Cells.

Cell Line	Condition	No. of Replicates[a]	Mean	Standard Deviation	Fold Increase[b]
HCT116	siControl	6	0.418	0.059	N/A
	si*SMC1A*-P	6	3.487	1.047	8.34
	si*SMC2*-P	6	4.082	2.309	9.77
	si*SMC4*-P	6	5.217	3.827	12.48
	si*NCAPD2*-P	6	3.442	1.190	8.23
	si*NCAPD3*-P	6	0.990	0.313	2.37
	si*NCAPH*-P	6	1.735	0.664	4.15
	si*NCAPH2*-P	6	2.552	2.044	6.11
	si*NCAPG*-P	6	3.893	7.296	9.31
	si*NCAPG2*-P	6	1.567	0.865	3.75

[a]≥100 nuclei/replicate analyzed.
[b]Fold increase in mean MN formation relative to siControl (not applicable; N/A).

Table S3: *SMC2* Silencing Corresponds with Significant Increases in the Cumulative NA Distribution Frequencies in HCT116.

Cell Line	Condition	N[a]	Mean	Standard Deviation	Fold Increase[b]	CDF p^c
HCT116	siControl	200	158.9	43.3	N/A	N/A
	si*SMC2*-2	200	266.9	117.6	1.7	<0.0001
	si*SMC2*-3	200	248.5	95.3	1.6	<0.0001
	si*SMC2*-P	200	339.2	136.0	2.1	<0.0001

[a]Number (N) of nuclei analyzed.
[b]Fold increase in mean NA relative to siControl (not applicable; N/A).
[c]Two sample KS test assessing differences in the cumulative distribution frequencies of NAs relative to siControl (N/A, not applicable); $p = <0.01$ is considered statistically significant.

Table S4: *SMC2* Silencing Corresponds with Significant Increases in the MN Formation in HCT116.

Cell Line	Condition	No. of Replicates[a]	Mean	Standard Deviation	Fold Increase[b]
HCT116	siControl	3	0.4333	0.0850	N/A
	si*SMC2*-2	3	5.3967	2.6799	12.4
	si*SMC2*-3	3	4.1900	3.7280	9.7
	si*SMC2*-P	3	4.8333	1.6494	11.2

[a]≥100 nuclei/replicate analyzed.
[b]Fold increase in mean MN formation relative to siControl (not applicable; N/A).

Table S5: Condensin Gene Silencing Induces Significant Changes in Cumulative Frequency Distributions for Chromosome Numbers in HCT116 Cells.

Cell Line	Condition	N[a]	Percentile			Range	CDF
			25th	50th	75th		*p*[b]
HCT116	siControl	100	45	45	45	41-90	N/A
	si*SMC2*-2	119	40	43	50	35-164	<0.0001
	si*SMC2*-3	112	39	42	51	32-166	<0.0001
	si*SMC2*-P	101	38	41	44	31-148	<0.0001

[a]Number (N) of enumerable mitotic chromosome spreads.
[b]Two sample KS tests assessing differences in the cumulative distribution frequencies of chromosome numbers relative to siControl (N/A, not applicable); p = <0.01 is considered statistically significant.

Table S6: Condensin Gene Silencing Induces Significant Changes in Cumulative Frequency Distributions for Chromosome Numbers in HCT116 Cells.

Cell Line	Condition	N[a]	Percentile			Range	CDF
			25th	50th	75th		p[b]
HCT116	siControl	100	45	45	45	43-92	N/A
	si*SMC2*-P	60	38	41	44	31-148	<0.0001
	si*SMC4*-P	53	38	41	43	32-82	<0.0001
	si*NCAPD2*-P	100	43	45	45	37-199	0.0008
	si*NCAPD3*-P	98	43	45	46	35-128	<0.0001
	si*NCAPH*-P	100	44	45	48	33-109	<0.0001
	si*NCAPH2*-P	89	45	45	47	36-198	0.0006
	si*NCAPG*-P	100	44	46	49	32-93	<0.0001
	si*NCAPG2*-P	91	45	46	48	38-118	<0.0001

[a]Number (N) of enumerable mitotic chromosome spreads.
[b]Two sample KS tests assessing differences in the cumulative distribution frequencies of chromosome numbers relative to siControl (N/A, not applicable); p = <0.01 is considered statistically significant.

Table S7: Condensin Gene Silencing Corresponds with Significant Increases in the Cumulative Nuclear Area Distribution Frequencies in hTERT Cells.

Cell Line	Condition	N[a]	Mean	Standard Deviation	Fold Increase[b]	CDF *p*[c]
hTERT	siControl	1947	231.1	67.1	N/A	N/A
	si*SMC1A*-P	705	301.6	85.8	1.31	<0.0001
	si*SMC2*-P	1338	278.6	80.8	1.21	<0.0001
	si*SMC4*-P	2675	225.8	75.4	0.98	<0.0001
	si*NCAPD2*-P	2060	245.9	77.7	1.06	<0.0001
	si*NCAPD3*-P	2479	246.8	75.3	1.07	<0.0001
	si*NCAPH*-P	1812	258.4	75.8	1.12	<0.0001
	si*NCAPH2*-P	1770	241.8	74.1	1.05	<0.0001
	si*NCAPG*-P	2291	233.1	72.5	1.01	0.0711
	si*NCAPG2*-P	2118	243.2	74.5	1.05	<0.0001

[a]Number (N) of nuclei analyzed.
[b]Fold increase in mean NA relative to siControl (not applicable; N/A).
[c]Two sample KS tests assessing differences in the cumulative distribution frequencies of NAs relative to siControl (N/A, not applicable); $p = <0.01$ is considered statistically significant.

Table S8: Condensin Gene Silencing Induces Increases in the MN Formation in hTERT Cells.

Cell Line	Condition	No. of Replicates[a]	Mean	Standard Deviation	Fold Increase[b]
hTERT	siControl	6	0.978	0.364	N/A
	si*SMC1A*-P	6	1.798	1.148	1.84
	si*SMC2*-P	6	3.358	0.804	3.43
	si*SMC4*-P	6	1.598	0.618	1.63
	si*NCAPD2*-P	6	1.477	0.640	1.51
	si*NCAPD3*-P	6	2.730	1.234	2.79
	si*NCAPH*-P	6	0.832	0.579	0.85
	si*NCAPH2*-P	6	1.240	0.556	1.27
	si*NCAPG*-P	6	1.163	0.316	1.19
	si*NCAPG2*-P	6	2.382	1.444	2.44

[a]≥100 nuclei/replicate analyzed.
[b]Fold increase in mean MN formation relative to siControl (not applicable; N/A).

Table S9: Condensin Gene Silencing Induces Increases in the MN Formation in hTERT Cells.

Cell Line	Condition	N[a]	Percentile 25th	Percentile 50th	Percentile 75th	Range	CDF *p*[b]
hTERT	siControl	100	46	46	46	41-92	N/A
	si*SMC2*-P	17	46	48	55	32-88	<0.0001
	si*NCAPD3*-P	100	46	50	53	38-104	<0.0001
	si*NCAPG2*-P	100	45	46	51	31-98	<0.0001

[a]Number (N) of enumerable mitotic chromosome spreads.
[b]Two sample KS tests assessing differences in the cumulative distribution frequencies of chromosome numbers relative to siControl (N/A, not applicable); p = <0.01 is considered statistically significant.
